# A Cascaded Dual Spiral Microfluidic Chip for Continuous Separation of Multicomponent Microparticles

**DOI:** 10.3390/mi17040469

**Published:** 2026-04-13

**Authors:** Renxuan Zhang, Ting Liu, Jianlong Zhao, Gaozhe Cai

**Affiliations:** 1 School of Microelectronics, Shanghai University, Shanghai 200444, China; 2 State Key Laboratory of Transducer Technology, Shanghai Institute of Microsystem and Information Technology, Chinese Academy of Sciences, Shanghai 200050, China; 3 Shanghai Frontier Innovation Research Institute, Shanghai 201108, China

**Keywords:** inertial microfluidics, cascaded microchannels, passive flow resistance matching, continuous particle sorting, sheathless, label-free

## Abstract

Inertial microfluidics is promising for the high throughput, label-free continuous separation of multicomponent microparticles. However, conventional single spiral microchannels struggle to separate three or more particle types, while traditional cascaded systems relying on sheath fluids or multiple pumps suffer from increased operational complexity. To address this, we propose a cascaded dual spiral microfluidic chip based on passive flow resistance matching. Driven by a single syringe pump without sheath flow, it achieves continuous sorting of three particle types. An adaptive flow resistance network is incorporated: the first stage channel maintains high velocity to preferentially extract large particles via strong inertial lift forces. The fluid then enters the second stage through a predetermined geometric resistance for automatic deceleration. Experiments demonstrate that at 1.6 mL/min, the system achieves continuous separation of a 1:10:10 mixture of 15, 10, and 5 µm microparticles. The 15 µm target recovery rate reaches 92%, while the collection purities for 10 µm and 5 µm particles exceed 98% and 99%, respectively. This purely passive fluidic architecture simplifies cascaded sorting, providing a robust engineering solution for complex multicomponent sample preprocessing.

## 1. Introduction

The continuous separation of complex multicomponent microparticles by size holds significant application value in fields such as biomedical diagnostics, environmental monitoring, and industrial powder classification [1,2]. Traditional centrifugation or microporous filtration methods typically suffer from inherent drawbacks, including susceptibility to clogging, limited throughput, and vulnerability to causing mechanical damage to microparticle samples [3,4,5]. In recent years, microfluidic technology has demonstrated precise fluid manipulation capabilities at the microscale. Consequently, it has provided an ideal platform for sorting microparticles at high throughput without the use of labels [6,7,8].

Among various microfluidic sorting technologies, inertial microfluidics based on spiral microchannels has garnered significant attention. This is due to its simple structure, high processing throughput, and ease of seamless integration [9,10]. In curved microchannels, the fluid generates a secondary flow perpendicular to the main flow direction (i.e., Dean vortex) due to centrifugal effects. Microparticles flowing through the channel undergo regular lateral migration under the combined action of Dean drag forces and shear-induced inertial lift forces. Ultimately, given appropriate geometric and fluidic parameters, they form stable focusing streamlines at specific transverse equilibrium positions [11]. This technology has been widely proven capable of efficiently separating sample mixtures with a sufficient size difference [12,13].

However, complex samples in practical applications often contain three or more components with similar sizes. Achieving the simultaneous separation of microparticles of multiple sizes in a conventional single spiral channel poses a severe fluid dynamic challenge. Specifically, the inertial lift force exerted on a microparticle is proportional to the fourth power of its diameter, whereas the Dean drag force is only proportional to its diameter [14]. At relatively high flow rates, large microparticles (e.g., 15 µm) are absolutely dominated by the inertial lift force, enabling them to rapidly form stable focusing near the inner wall of the channel. In contrast, for medium and small microparticles (e.g., 10 µm and 5 µm), the inertial lift force they experience attenuates sharply as their size decreases. Consequently, their trajectories become more susceptible to dispersion caused by the perturbation of the secondary Dean flow. Therefore, under the same flow rate conditions, the spatial streamlines of medium and small microparticles are highly prone to overlapping. This overlap makes it difficult for a single flow field structure to simultaneously achieve the efficient focusing and separation of microparticles of multiple sizes.

To overcome the theoretical bottlenecks of single channels, researchers have proposed the concept of cascaded microfluidics, which aims to achieve separation across multiple stages by connecting channels with different geometric characteristics in series [15,16,17]. In existing cascaded microfluidic studies, the serial connection of multiple separation modules alters the internal pressure distribution. Consequently, additional buffer inlets are typically introduced at the cascade nodes for active flow rate compensation. However, incorporating multiple fluid inlets increases the complexity of the system. Furthermore, determining the final operating parameters often relies on preliminary experimental assessments of individual modules, followed by flow velocity simulations and extensive experimental parameter sweeping to identify the optimal flow rate combination. This essentially constitutes an empirical optimization process. Additionally, the introduction of sheath flow inevitably dilutes the initially rare target samples, which may limit its translational application in practical preprocessing [18,19,20].

To address these challenges in fluid dynamics and system integration, this study proposes a single inlet cascaded dual spiral microfluidic chip based on passive hydrodynamic resistance matching. In contrast to systems that rely on multiple pumps for active compensation, our design provides a deterministic and quantitative approach to fluid control. The system is driven entirely by a single syringe pump and eliminates the need for sheath flow. This approach significantly reduces the reliance on peripheral hardware, decreasing the required pumping equipment by more than half. The ratio of flow splitting at the outlet of the first spiral stage is passively governed by the relative hydrodynamic resistances of the downstream branches. The resistance network, designed in advance, enables subsequent spiral channels to achieve their respective target flow rates seamlessly. This approach eliminates the uncertainties and operational complexities typically associated with balancing sample and sheath flows.

The optimal operating flow rates for individual spiral stages are first determined through particle trajectory experiments in single spiral microchannels across a range of flow rates. These results subsequently guide the design of the hydrodynamic resistance network. During the design phase of the cascaded spiral microfluidic chip, analytical predictions of the flow splitting ratios are achieved through preliminary calculations. Subsequently, numerical simulations are employed to cross validate the global steady state flow field and the resulting flow splitting ratios. Therefore, this methodology facilitates a transition from empirical trial-and-error to quantitative forward design, improving both the theoretical rigor and the engineering transferability of the device. Experimental results demonstrate that at an inlet flow rate of 1.6 mL/min, the system successfully separated mixed 15 µm, 10 µm, and 5 µm polystyrene microparticles with an initial concentration ratio of 1:10:10. The first stage high velocity channel achieved a 92% recovery rate for the enrichment of large microparticles (15 µm). The second stage channel then utilized the passively attenuated flow field to extract the 10 µm and 5 µm background microparticles, achieving collection purities exceeding 98% and 99%, respectively. This single inlet cascaded dual spiral microfluidic chip based on passive hydrodynamic resistance matching effectively lowers the hardware threshold for multi stage sorting systems, providing a reliable and transferable engineering solution for the continuous separation of complex microparticles.

## 2. Materials and Methods

### 2.1. Chip Design and Working Principle

**Figure 1:**
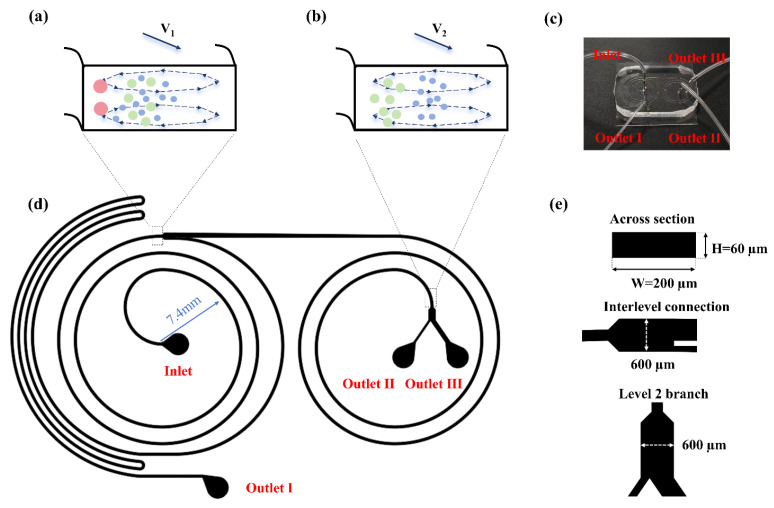
Schematic diagram of the geometric structure and characteristic dimensions of the cascaded dual spiral microfluidic chip. (**a**) Schematic diagram of the first stage end situation, with 15 µm, 10 µm, and 5 µm particles shown in red, green, and blue respectively; (**b**) Schematic diagram of the second stage end situation; (**c**) Image of the chip, including one sample inlet and three outlets; (**d**) Overall top-down schematic of the dual spiral design; (**e**) Schematic diagram of the cross section of the helical channel, the inter-stage connection, and the level 2 branch dimensions.

Figure 1 illustrates the architecture of the cascaded microfluidic chip developed in this study. The device integrates two consecutive spiral microchannels on a single plane, featuring one inlet and three separate outlets dedicated to collecting 15 µm, 10 µm, and 5 µm microparticles, respectively. To prevent physical clogging of sample particles within the spiral inertial microfluidic channel while ensuring that the blockage ratio *a/D_h_* > 0.07 for both the 15 µm and 10 µm particles, where *D_h_* is the hydraulic diameter of the channel defined as *a*/*D_h_* = *2wh*/(*w* + *h*), the rectangular cross section of the spiral channel is designed with a width (*w*) of 200 µm and a uniform height (*h*) of 60 µm. Each spiral channel consists of 2 loops, featuring a minimum radius of curvature (*R*) of 7.4 mm. This specific design provides sufficient effective focusing length for stable particle sorting. Simultaneously, it avoids excessive fluidic resistance in the single stage spiral, thereby preventing a massive total system resistance after cascading. Spatially, the system adopts a reversed configuration. The fluid in the initial channel spirals outwards from the center. Its outer outlet then seamlessly joins the outer inlet of the subsequent channel on the same horizontal plane, directing the fluid to spiral inwards in the opposite direction. Furthermore, localized widening and asymmetric bifurcation structures are incorporated at the connection between the two stages as well as at the terminus of the final spiral. The inner branch located at the bifurcation between the stages is used for the collection of 15 µm microparticles. This branch features an elongated geometry to increase fluidic resistance (Section 3.2).

Spiral microchannels are among the most commonly used core structures for continuous microparticle separation [21,22,23]. In spiral inertial microfluidic channels, the lateral migration behavior of microparticles is primarily governed by the interaction between the net inertial lift force (*F_L_* ∝ *a*^4^) and the Dean drag force (*F_D_* ∝ *a*) induced by the secondary flow [24,25,26,27]. The magnitude of the inertial lift force *F*_L_ is proportional to the fourth power of the microparticle diameter and the square of the maximum fluid velocity, expressed as follows [28,29,30,31]:

(1) FNet,L=fLRe,x/hρUm2a4Dh2

where ρ is the fluid density, *U*_m_ is the maximum velocity at the channel center (for a rectangular channel, typically defined as *U*_m_ = 1.5 *U*, where *U* is the average fluid velocity), a is the microparticle diameter, and *f*_L_ is the lift coefficient dependent on the microparticle position and the channel Reynolds number.

Additionally, at elevated Reynolds numbers (*Re*), a secondary flow directed perpendicular to the primary streamline emerges within the curved channel. This motion, known as Dean flow, is driven by the spatial variation in fluid inertia [32,33,34]. The secondary Dean flow manifests as a pair of symmetric vortices rotating in opposite directions within the upper and lower regions of the channel cross section. The intensity of the Dean flow can be evaluated by the dimensionless Dean number (*De*):

(2) $$De={\left(\frac{D_{h}}{2R}\right)}^{1/2}Re$$

This secondary flow exerts a Dean drag force (*F*_D_) on suspended microparticles, and its approximate calculation formula is [33,35]:

(3) $$F_{D}∼5.4\times 10^{-4}\pi \mu D_{e}^{1.63}a$$

Here, *µ* denotes the dynamic viscosity of the fluid. The Dean drag force entrains particles into the symmetrically rotating vortex streamlines, while the inertial lift force tends to confine the particles to specific equilibrium positions within the cross section. Consequently, the relative magnitude of these two forces dictates the final dynamic behavior of the particles. A dimensionless parameter *R*_f_ can be introduced to characterize the competition between the inertial lift force and the Dean drag force. This parameter integrates the particle blockage ratio and the channel curvature characteristics.

(4) $$R_{f}=\frac{2a^{2}R}{D_{h}^{3}}>0.08$$

Research indicates that when *R*_f_ satisfies the aforementioned condition, the inertial lift force can effectively overcome the Dean drag force to achieve particle focusing. The calculated *R*_f_ values for both the 15 µm and 10 µm particles are strictly greater than 0.08, with the 15 µm particles exhibiting an even larger *R*_f_. This verifies that the designed channel cross section guarantees the effective focusing of both particle sizes. Furthermore, it dynamically explains why the 15 µm particles establish their focusing equilibrium position closer to the inner wall of the spiral channel [36,37].

The migration mechanisms of the three particle sizes within the microchannel exhibit significant differences. Because the 5 µm particles struggle to maintain an efficient focusing state, their divergent trajectories induced by the secondary flow perturbation easily overlap spatially with the focusing streamlines of other particles, particularly the 10 µm particles. Consequently, a single spiral channel cannot achieve the simultaneous and efficient separation of these three particle types. Additionally, microparticles smaller than 5 µm similarly struggle to maintain effective inertial focusing and may contaminate the sorting purity of the 15 µm particles. Consequently, the lower size limit of microparticles that this spiral microchannel can effectively process is approximately 5 µm.

Based on this theoretical rationale, we propose a cascaded dual spiral structure. A high flow velocity is set in the first stage channel to preferentially and stably separate the 15 µm particles, which are dominated by a strong inertial lift force. Subsequently, the mixture containing the 10 µm and 5 µm particles is directed into the second stage channel. In the second stage, the flow velocity is automatically reduced through passive flow resistance matching. Because the inertial lift force attenuates with the square of the flow velocity (*F*_L_ ∝ *U*^2^), while the Dean drag force attenuates more gradually (*F*_D_ ∝ *U*), and the curvature of the spiral channel continuously increases from the outside to the inside, the force ratio (*F*_L_/*F*_D_) decreases significantly. This shift in the mechanical mechanism effectively weakens the influence of the inertial lift force exerted on the small particles (5 µm) and enhances the dominant role of the Dean secondary flow in their lateral migration. Combined with the widened outlet design at the terminus of the channel, the geometric divergence effect of the streamlines is utilized to moderately amplify the lateral distance between the 10 µm and 5 µm particles. This ultimately achieves highly efficient ternary separation of the complex mixture.

**Figure 2:**
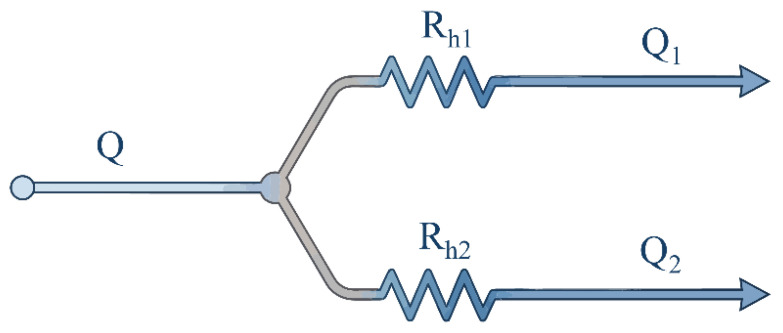
Schematic diagram of the equivalent flow resistance network physical model at the bifurcation connections in the cascaded microfluidic chip.

In a microscale laminar flow system, the flow distribution behavior within a bifurcated network can be analogized to the classic electrical Ohm’s law (as shown in Figure 2) [38]. When a fluid with a volumetric flow rate *Q* flows through a microchannel with a pressure drop of ∇*P* across its ends, the equivalent fluidic resistance (*R*_h_) satisfies the following relationship:

(5) $$Q=\frac{\Delta P}{R_{h}}$$

The asymmetric bifurcation at the junction between stages is hydrodynamically equivalent to a parallel circuit. Given that all chip outlets are maintained at atmospheric pressure, the pressure drop across these parallel branches is identical. Consequently, the flow distribution ratio at the bifurcation is determined by the inverse proportion of the fluidic resistance between the two paths [39,40]:

(6) $$\;\frac{Q_{1}}{Q_{2}}=\frac{R_{h2}}{R_{h1}}$$

(7) $$R_{h}\approx \frac{12\mu L}{wh^{3}\left[1-\frac{192h}{\pi^{5}w}\tanh\left(\frac{\pi w}{2h}\right)\right]}$$

The fluidic resistance of each branch can be calculated using the Hagen-Poiseuille approximation equation for rectangular channels [41]. Given a constant channel height, the fluidic resistance (*R_h_*) is primarily determined by the physical channel width (*W*) and the flow length (*L*). tanh is the hyperbolic tangent function, which is used to correct for the viscous drag at the edges of the rectangular cross-section. When the sample flows through the first stage channel, larger microparticles flow along the inner wall and enter the inner high resistance branch, while smaller microparticles enter the second stage channel along with the majority of the fluid. By adjusting the geometric dimensions of the inner elongated branch to preset the resistance ratio, the system can adaptively and intuitively regulate the flow distribution between the two branches. In Section 3.1, the flow splitting ratio at the bifurcation is determined based on the optimal operating flow rates of the single spiral channels. Once this preset flow splitting ratio is established, the fluidic resistance (*R*_h_) ratio between the 15 µm collection branch and the second-stage inlet branch can be determined (Section 3.2). This achieves passive flow deceleration without the need for external pump or valve interventions.

### 2.2. Fabrication of the Chip

Standard soft lithography was employed for the high precision fabrication of the microfluidic chip. Initially, SU-8 negative photoresist was deposited onto the surface of a clean monocrystalline silicon wafer through spin coating. Following standard photolithography procedures comprising soft baking, ultraviolet (UV) exposure, post-baking, and development, an SU-8 master mold with a target channel height of 60 µm was successfully produced. Subsequently, a polydimethylsiloxane (PDMS) prepolymer and its curing agent were thoroughly mixed at a mass ratio of 10:1. The mixture was then placed in a vacuum desiccator for degassing to completely remove internal microbubbles. The degassed PDMS mixture was smoothly poured over the SU-8 mold and cured in an oven at 65 °C for 2 h. After curing, the PDMS replica containing the microchannel pattern was carefully peeled off the silicon template. A dedicated hole puncher was used to punch access ports at the predesigned fluid inlet and collection outlets. Finally, the punched PDMS chip and a clean glass slide were placed into an oxygen plasma cleaner for surface activation. Immediately following the treatment, the two components were brought into contact. Uniform pressure was then applied to achieve permanent covalent bonding between the PDMS chip and the glass substrate.

### 2.3. Sample Preparation

To physically simulate the extreme disparities in size and concentration found in complex real samples, standard polystyrene fluorescent microspheres (Huge Biotechnology, Shanghai, China) were employed as the test model for the multicomponent suspension system. Particles with diameters of 15 µm (green fluorescence), 10 µm (red fluorescence), and 5 µm (blue fluorescence) were utilized in the experiments. Regarding the concentration gradient, the working concentration of the 15 µm particles, which simulated large-sized rare targets, was set to 5 × 10^4^ particles/mL. Meanwhile, the concentrations of the 10 µm and 5 µm particles, representing the background population, were both set to 5 × 10^5^ particles/mL. This design strictly maintained the initial concentration ratio of the mixed system at 1:10:10. To prevent the physical aggregation of the polystyrene particles, the sample suspensions were uniformly prepared in 1 × phosphate-buffered saline (PBS). Tween-20 with a volume fraction of 0.1% (*v*/*v*) was added to effectively reduce the fluidic surface tension and inhibit non-specific adsorption. As a highly diluted aqueous solution, its hydrodynamic properties are nearly identical to those of pure water at room temperature. Specifically, the dynamic viscosity is approximately 1.0 × 10^−3^ Pa·s, and the fluid density is about 1000 kg/m^3^. In terms of chemical properties, 1 × PBS maintains a stable physiological pH of 7.4 and a conductivity of approximately 16 mS/cm. Prior to each sample injection, the centrifuge tube containing the mixture was placed in an ultrasonic water bath for 5 min of sonication, ensuring that particles of all sizes entered the microfluidic system in a completely monodispersed state.

### 2.4. Numerical Simulation Setup

Three dimensional finite element numerical simulations of the chip were conducted to verify the flow splitting ratio within the cascaded fluidic resistance network. These simulations were performed using the COMSOL Multiphysics software (version 6.3) package. The Laminar Flow physics interface was employed, and the fluid medium was set as incompressible deionized water at room temperature (density ρ = 998.2 kg/m^3^, dynamic viscosity µ = 1.003 mPa·s). Regarding the boundary conditions: a specified volumetric flow rate was applied at the microchannel inlet; all terminal outlets of the cascaded system were set to a zero relative static pressure boundary for free outflow; and a no-slip boundary condition was applied to all internal physical walls of the chip. To accurately resolve the Dean vortex boundary layers induced by the curved channels and the steep velocity gradients near the walls, boundary layer mesh refinement was implemented across the entire wall region. Finally, an optimized mesh configuration comprising approximately 2.03 million elements was utilized for the steady-state flow field solver, striking an optimal balance between computational accuracy and resource consumption.

### 2.5. Experimental Setup and Data Quantification

In the microfluidic sorting experiments, the prepared mixed microparticle suspension was loaded into a standard medical syringe. It was then continuously injected into the cascaded microfluidic chip at a constant flow rate using a high-precision micro-syringe pump (LSP02 2B, Longer Precision Pump, Baoding, China). The real-time experimental observation platform primarily consisted of an inverted fluorescence microscope (ICX41, SOPTOP, Ningbo, China) equipped with a high-sensitivity camera (M3LY500TFS, LITO, Shenzhen, China). This setup was used to record the high speed fluorescent motion trajectories of the microparticles at various positions within the cascaded microchannels. These dynamic processes are provided as Supplementary Materials. The captured multi-channel raw fluorescence images were subsequently imported into ImageJ software (version 1.53) for quantitative analysis. The system extracted the spatial distribution curves of relative fluorescence intensity. By calculating the centroid coordinates and full width at half maximum (FWHM) of the primary peaks, the lateral focusing positions and focusing band widths were accurately quantified. All flow rate conditions were maintained for more than 2.5 min, and observation was carried out after the particle trajectory stabilized. For the final evaluation of the sorting performance, the effluent from each chip outlet was collected, and the target microparticles were physically counted using a hemocytometer (Shanghai Qiujing, Shanghai, China) under a microscope. The sorting efficacy of the system was quantified using two core metrics: separation purity and recovery rate. Purity is defined as the percentage of target sized microparticles in a specific outlet relative to the total population collected in that outlet. The recovery rate is defined as the percentage of target sized microparticles collected in a specific outlet relative to the total amount of those particles injected into the inlet. To ensure the reliability and reproducibility of the results, all microfluidic sorting experiments were independently repeated at least three times (*n* = 3). Quantitative data from repeated experiments are presented as the mean ± standard deviation (SD).

## 3. Results and Discussion

### 3.1. Lateral Migration in a Single Spiral Channel

A standalone spiral microchannel with a cross section identical to that of the cascaded device was initially utilized as a preliminary test platform. This setup was used to characterize the inertial focusing behavior of multicomponent microparticles and identify the optimal separation parameters. The experimental system examined the spatial migration behavior of 15 µm, 10 µm, and 5 µm fluorescent particles at the terminal end of the channel. The inlet driving flow rates for these tests ranged from 0.1 mL/min to 1.8 mL/min.

**Figure 3:**
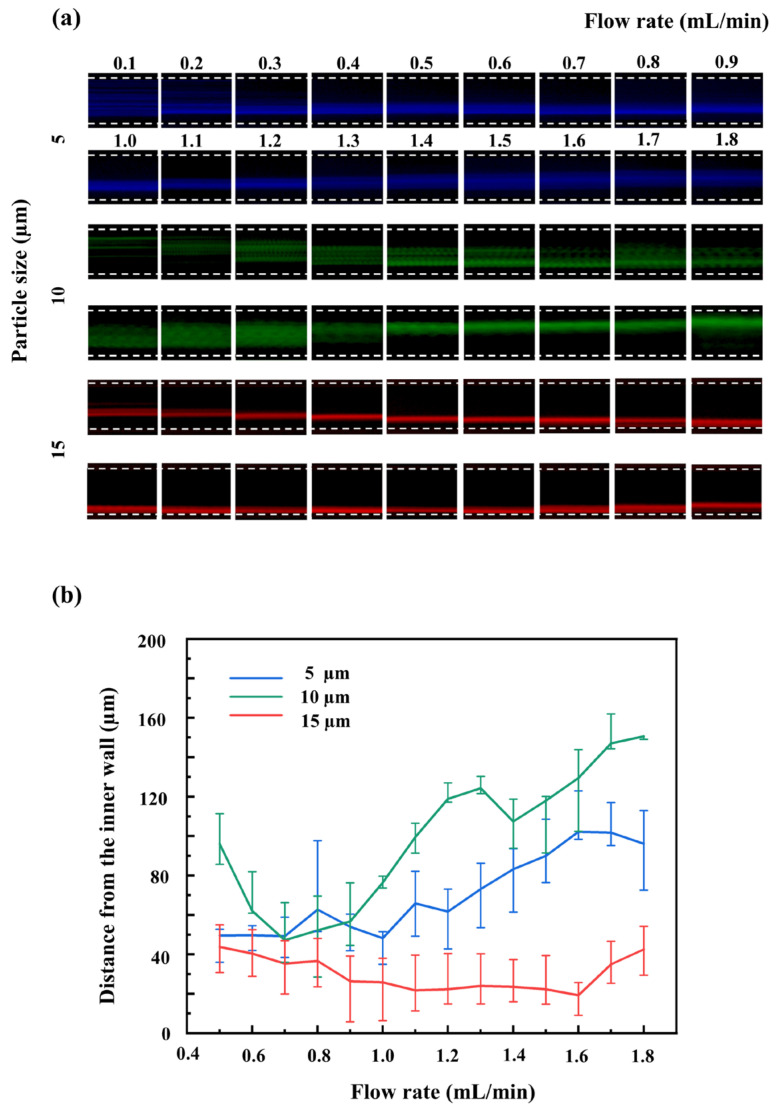
(**a**) Fluorescence trajectory evolution of microparticles of different sizes (15 µm, 10 µm, 5 µm) at various inlet flow rates in the first-stage channel; (**b**) Quantitative relationship between the lateral centroid positions of the microparticles and the inlet flow rates.

In the first stage channel, suspensions of 15 µm, 10 µm, and 5 µm fluorescent polystyrene microspheres were pumped into the test chip, with an inlet flow rate gradient set from 0.1 to 1.8 mL/min (Figure 3a). The 15 µm and 10 µm microparticles satisfied the critical focusing threshold of *a*/*D_h_* > 0.07 and could thus form distinct focusing bands within the flow field [24]. Since the physical ratio of the inertial lift force to the Dean drag force is proportional to the third power of the particle diameter (*F*_L_/*F*_D_ ∝ *a*^3^), the inertial lift force exerted on the 15 µm microparticles was absolutely dominant. Consequently, their focusing centroids rapidly migrated toward the inner wall of the channel, exhibiting a highly focused state near the inner wall. The force ratio for the 10 µm microparticles was relatively small; at flow rates exceeding 1.4 mL/min, their equilibrium positions stably distributed in an outer region slightly farther from the inner wall [37]. In contrast, the physical size of the 5 µm microparticles had not reached the critical condition for strong inertial focusing. At lower flow rates, the 5 µm microparticles experienced minor flow field perturbations, resulting in relatively narrow distribution bands. However, as the flow rate continuously increased, the curvature-induced secondary Dean flow within the channel intensified sharply [36]. Because the inertial lift force on the 5 µm microparticles was relatively small, they could not resist the substantial Dean drag force perturbations at high flow rates. As a result, the particles were entrained by the vortices and dispersed across the cross section. This led to a significant increase in the trajectory distribution band width.

The spatial distribution profiles in Figure 3b illustrate the migration of the three microparticle types. At an inlet flow rate of 1.6 mL/min, the 15 µm microparticles formed a relatively narrow focusing band close to the inner wall. This created a sufficiently wide physical gap between the 15 µm particles and the 10 µm and 5 µm microparticles, which were distributed toward the outer region of the channel. At this specific flow rate, the spatial overlap between large particles and both medium and small particles reached a minimum, providing a sound basis for effective separation. Consequently, 1.6 mL/min was established as the optimal operational flow rate for the initial spiral channel in this study. However, under this specific flow condition, the spatial trajectories of the 10 µm and 5 µm microparticles exhibit significant overlap, precluding effective separation. Because a single high velocity flow field is insufficient to simultaneously separate all three particle sizes, the remaining mixture must be directed into the subsequent cascaded channel for further processing.

**Figure 4:**
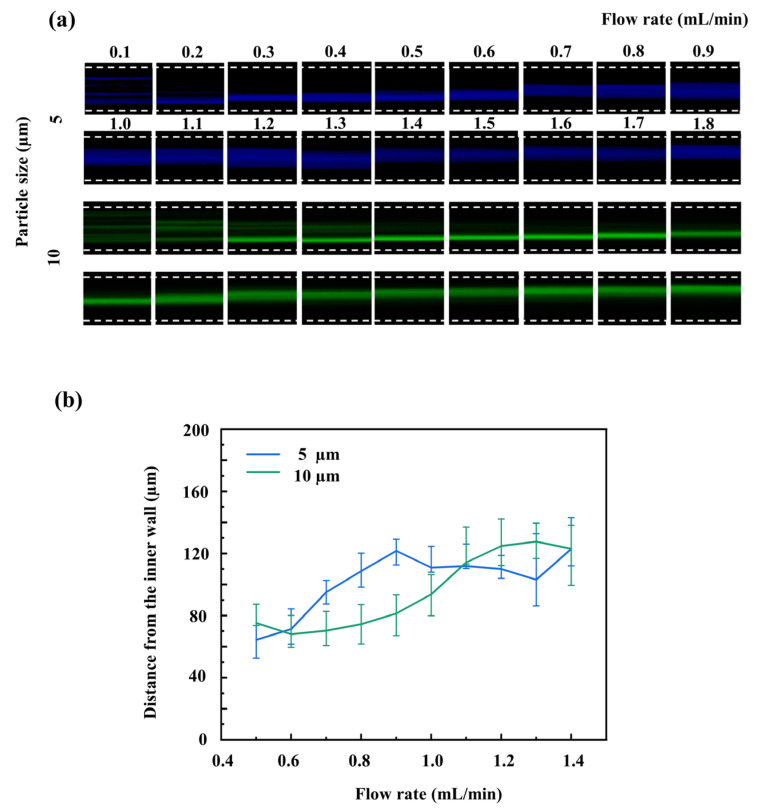
(**a**) Evolution of fluorescence trajectories for medium and small microparticles (10 µm and 5 µm) across a range of inlet flow rates within the second stage; (**b**) Quantitative relationship between the lateral centroid positions of the medium and small microparticles and the corresponding inlet flow rates.

The 10 µm and 5 µm microparticles could not be differentiated in the first stage channel. Therefore, the single spiral channel of the second stage was further tested to investigate the lateral migration behavior of microparticles at low flow rates. Only these two types of microparticles were introduced into the system. The inlet flow rate gradient remained set from 0.1 to 1.8 mL/min. Quantitative experimental data demonstrate that the lateral migration positions of the microparticles were highly sensitive to flow rate variations (Figure 4a). At lower flow rates, both the 5 µm and 10 µm microparticles were distributed toward the inner side of the channel, spatially overlapping each other. As the flow rate gradually increased, the secondary vortex effect in the flow field strengthened, causing both sizes of microparticles to shift toward the outer side of the channel. During this dynamic evolution process, because the 10 µm microparticles were still constrained by the inward inertial lift force, their outward shifting velocity was relatively slow. In contrast, the 5 µm microparticles were primarily dominated by the Dean drag force, resulting in a much more pronounced outward migration [30]. This mechanical difference in lateral shifting rates gradually generated and widened the separation gap between the two (Figure 4b). When the flow rate further increased, both types of microparticles continued to move toward the outer wall, causing their distribution areas to overlap again and the separation gap to decrease. Ultimately, this study determined 0.8 to 0.95 mL/min as the optimal separation working flow rate range for the second stage spiral microchannel. At these specific flow rates, the centroid distance between the 10 µm and 5 µm microparticles reached a relative maximum. This established an optimal hydrodynamic foundation to further amplify their physical separation using an expanded channel geometry, ultimately enabling collection with high purity.

### 3.2. Quantitative Design of the Flow Resistance Network

As previously described, the optimal operating flow rates for the first and second stage spiral channels were determined to be 1.6 mL/min and 0.9 mL/min, respectively. To achieve this flow distribution, an elongated geometric structure was adopted for the inner 15 µm collection branch at the bifurcation. For this elongated structure, its cross-sectional shape and area were fixed to be identical to those of the main spiral channel. During the branch design, it was arranged to wrap along the first stage spiral channel. Its channel length served as the primary adjustable parameter until the cascaded device could achieve the same hydrodynamic characteristics as the individual components.

According to Equation (6), the flow distribution at the bifurcation is proportional to the reciprocal of the fluidic resistances of the two downstream branches. To reach the optimal target flow rates, the ideal fluidic resistance ratio between the 15 µm collection branch and the second stage inlet branch should be approximately 1.29. This provided a quantitative guiding principle for the subsequent design of the channel geometry.

Based on the Hagen Poiseuille approximation equation for rectangular channels (Equation (7)), we quantitatively evaluated the total hydraulic resistance of the second stage inlet branch (*R_h_*_2_). This branch comprises a tapered inter stage connection section (width linearly decreasing from 400 µm to 200 µm, length = 21.0 mm) and a continuous spiral section (width = 200 µm, centerline length = 114.9 mm). By applying Simpson’s rule for numerical integration on the tapered section, the total hydraulic resistance of this branch was determined to be 4.40 × 10^13^ Pa·s/m^3^.

Similarly, we evaluated the inner 15 µm collection branch (*R_h_*_1_). To ensure calculation accuracy, the model divided this collection branch into two segments: an initial narrow section (width = 100 µm, centerline length = 1.4 mm) and a subsequent widened section (width = 200 µm, centerline length = 174.4 mm). Since the fluid within the microchannel is strictly in the laminar regime, the local pressure drop losses caused by the channel width expansion are negligible. By applying the series resistance model, the precise hydraulic resistance of this branch was calculated to be 6.09 × 10^13^ Pa·s/m^3^.

In summary, the actual designed resistance ratio (*R_h_*_1_/*R_h_*_2_) of the system is approximately 1.38 (close to the expected design value of 1.29). According to Equation (6), this specific resistance ratio determines the precise flow distribution at the bifurcation: approximately 41.9% of the fluid is directed to the 15 µm collection outlet, while the remaining 58.1% enters the second stage. This means that under a total inlet flow rate of 1.6 mL/min, the actual operating flow rate of the second stage spiral channel is approximately 0.93 mL/min. This result highly aligns with the optimal target flow rate of 0.9 mL/min and falls well within the acceptable engineering error margin, fully demonstrating the rationality and success of the passive flow-matching design.

### 3.3. CFD Flow Field Simulation and Flow Split Validation

To validate the effectiveness of the aforementioned theoretical design, we investigated the flow velocity distribution across the entire integrated chip using COMSOL Multiphysics software. The preliminary calculation of hydrodynamic resistance using analytical formulas in the previous section provides a quantitative basis and initial guidance for our design. However, in an actual 3D spiral channel, the strong Dean vortices (secondary flow) induced by centrifugal forces result in additional kinetic energy dissipation. Therefore, the flow splitting ratio can be determined through three dimensional steady state flow field simulations. This approach provides a rigorous cross validation of the theoretical calculations.

**Figure 5:**
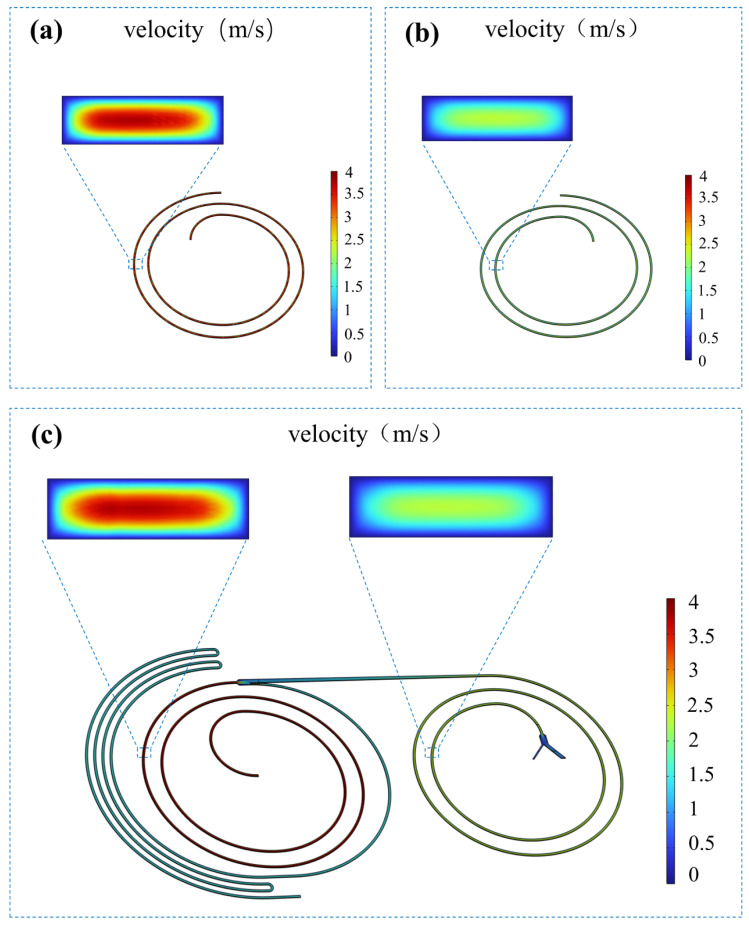
Flow rate simulation results executed by COMSOL Multiphysics. (**a**) Flow simulation of the first-stage independent spiral channel (velocity distribution corresponding to 1.6 mL/min); (**b**) Flow simulation of the second-stage independent spiral channel (velocity distribution corresponding to 0.9 mL/min); (**c**) Optimal flow velocity distribution in the cascaded dual spiral channel, demonstrating that the flow distribution at key nodes within the cascaded channel closely resembles the flow field characteristics of each independently channel.

As shown in the flow velocity distribution map (Figure 5c), the fluid maintained a relatively high velocity within the first stage spiral channel. The fluid experienced significant physical attenuation when reaching the inter stage widening region and the asymmetric bifurcation node. This effect was caused by the sudden increase in the channel cross sectional area combined with flow diversion through the resistance network.

To precisely quantify the flow field distribution ratio, surface integration function analysis was further employed based on the velocity simulation, successfully extracting the actual volumetric flow rates at each outlet cross-section.

(8) $$Q=\iint_{S}\left(u\cdot n_{x}+v\cdot n_{y}+w\cdot n_{z}\right)dS$$

where *Q* represents the volumetric flow rate of the branch and *S* denotes the outlet cross sectional area. The variables *u*, *v*, and *w* are the actual velocity components in the Cartesian coordinate system, while *n*_x_, *n*_y_, and *n*_z_ represent the components of the unit normal vector for the cross section.

At a total inlet driving flow rate of 1.6 mL/min, the fluid passed through the first stage bifurcation network. The inner high resistance collection branch (Outlet I) successfully diverted 0.695 mL/min, accounting for approximately 43.4% of the total system injection volume. Meanwhile, the remaining approximately 0.905 mL/min of fluid (accounting for 56.6%) was introduced into the second stage spiral channel. This proves that relying on the preset geometric structure ratio, the system can achieve precise flow rate deceleration without the intervention of external micro-pumps/valves or sheath fluids. After undergoing adaptive deceleration, the fluid flowed through the spiral channel in the second stage and bifurcated at the widened region near the terminus. The actual output volumetric flow rates of Outlet II and Outlet III were 0.191 mL/min and 0.714 mL/min, respectively. The sum of the flow rates from all branch outlets of the system is highly consistent with the total inlet flow rate, complying with the law of conservation of mass. It is worth noting that the simulated flow splitting ratio (43.4%) exhibits a slight deviation from the analytical prediction (41.9%). This negligible discrepancy is expected, as the 3D numerical model comprehensively accounts for the additional kinetic energy dissipation induced by the secondary Dean vortices, which the ideal Hagen-Poiseuille equation omits.

### 3.4. Sorting Performance of the Cascaded Chip

**Figure 6:**
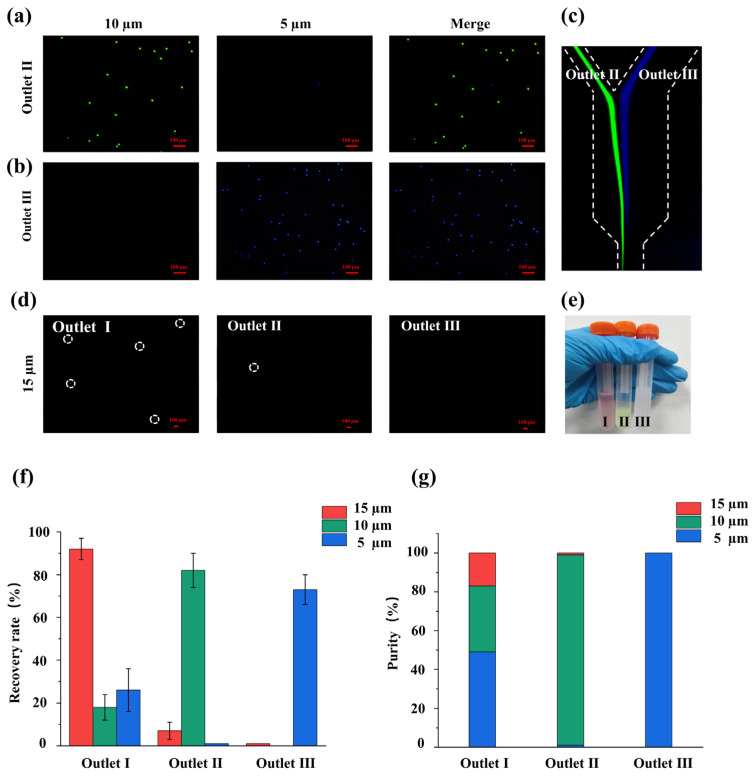
Mixed particles (15 µm, 10 µm, 5 µm) were used to test the cascade spiral microfluidic chip. (**a**) Fluorescence microscopy results of particles collected from Outlet II; (**b**) Fluorescence microscopy results of particles collected from Outlet III; (**c**) Fluorescence traces at Outlets II and III. (**d**) Distribution of 15 µm particles in the three outlets. (**e**) Comparison of physical samples collected from each outlet. Quantitative results of the sorting recovery rate (**f**) and purity (**g**) of the cascaded spiral microfluidic chip for three sizes of mixed microparticles (15 µm, 10 µm, 5 µm, with an initial ratio of 1:10:10).

Following the observation of flow field trajectories and the verification of flow distribution in the cascaded system, a quantitative analysis of the three way recovery rate and sorting purity of the chip was conducted. The total inlet flow rate of the chip was maintained at 1.6 mL/min. With an average fluid velocity exceeding 2 m/s, the entire separation process from initial injection to stable focusing and final outlet collection is accomplished within a residence time of merely tens of milliseconds. This highlights the continuous and ultra high throughput processing capabilities, achieving a total processing capacity of 1.0 × 10^8^ particles per hour, which is fundamentally crucial for practical applications. Once the system flow field stabilized, the effluents from the first stage 15 µm collection port (Outlet I), the second stage 10 µm collection port (Outlet II), and the 5 µm collection port (Outlet III) were collected at regular intervals. As shown in the fluorescence traces (Figure 6c), the majority of the 10 µm particles were directed to Outlet II, whereas the 5 µm particles predominantly flowed to Outlet III. This is further confirmed by the fluorescence microscopy images of the collected particles, which indicate that Outlet II mainly contains 10 µm particles (Figure 6a) and Outlet III mainly contains 5 µm particles (Figure 6b). Additionally, the fluorescence images of the 15 µm particles distributed across the three outlets demonstrate that they are primarily collected at Outlet I (Figure 6d). The physical sample suspensions collected from these three outlets are presented in Figure 6e.

An automated cell analyzer was used to perform concentration and particle size distribution statistics on the suspension samples collected from each outlet. These results were then compared with the particle concentrations of the initial mixed suspension. The measured concentration data were substituted into the previously defined evaluation metric calculation formulas. This allowed for the calculation of the recovery rates for the 15 µm, 10 µm, and 5 µm particles, along with the separation purities at their respective collection ports.

The statistical data results (Figure 6) show that the first stage separation port achieved a high recovery rate of 92.0% ± 5.5% for the 15 µm microparticles. The widening structure at the connection between the first and second stages effectively reduced the incorporation of impurity particles. The absolute concentration of the 15 µm particles in the collected fraction (Outlet I) is (1.06 ± 0.08) × 10^5^ particles/mL. Compared to the initial 5 × 10^4^ particles/mL, the concentration enrichment factor (CEF) is over 2 fold. The proportion of 15 µm particles increased from the initial 4.76% (1/21) to 17.3% ± 1.4%, yielding a purity enrichment factor (PEF) of approximately 3.6 fold. Consequently, the system successfully meets the criteria for first stage continuous pre enrichment, effectively extracting rare targets from an overwhelming background. The real time dynamic process and stable streamline formation of the 15 µm microparticles at the first stage bifurcation are demonstrated in.

In the second stage separation phase, the 10 µm and 5 µm microparticles produced significant differences in lateral shifting within the decelerated flow field. Combined with the widening structure before the terminal bifurcation of the second stage, the recovery rate of the 10 µm target microparticles in the second stage channel reached 82 ± 8.0%, and the collection purity of Outlet II was as high as 98.0 ± 0.8%. Simultaneously, Outlet III successfully directed the vast majority of the 5 µm background microparticles (recovery rate of 73.0 ± 7%) toward the waste terminal, with a removal purity reaching 99.0 ± 0.5%. Furthermore, the stable focusing behavior and continuous separation process of the microparticles at the terminus of the second stage are visually validated. The specific trajectories of the 10 µm and 5 µm microparticles are recorded in, respectively.

In summary, this cascaded microfluidic chip employs precise passive flow resistance matching and incorporates two widened channel geometries. This configuration successfully achieved the continuous separation and collection of three distinct microparticle populations. The device is driven entirely by a single pump, eliminating the need for external sheath fluid compensation. This performance fully validates the robust capability of the system to process complex samples containing particles of various sizes.

## 4. Conclusions

This study successfully developed a cascaded dual spiral microfluidic chip based on passive flow resistance matching. This architecture achieves the continuous and high purity size based sorting of multicomponent particles, specifically 15 µm, 10 µm, and 5 µm targets. Furthermore, the system completely eliminates the reliance of traditional multi stage microfluidic sorting on external sheath flow compensation and multiple independent pump valve setups. Driven solely by a single micro-syringe pump, it can process complex multicomponent fluid samples with a highly simplified hardware configuration.

The experimental results confirm the necessity of the adaptive deceleration mechanism. The highly efficient extraction of the large-sized 15 µm target relies on a high inlet flow rate of 1.6 mL/min, whereas the thorough separation of 10 µm and 5 µm particles necessitates reducing the flow rate to the 0.8–0.95 mL/min range. The chip successfully and passively reduced the equivalent flow rate entering the second stage main channel to 0.91 mL/min by presetting an asymmetric bifurcated flow resistance network. This effectively bridged the flow rate gap between the optimal separation conditions for particles of different sizes. Furthermore, the geometric widening structures designed at the inter stage connection and the end of the second stage significantly enlarged the lateral separation distance between particles, greatly reducing the risk of cross-contamination.

In the sorting performance evaluation, the system processed a complex mixed sample with an extremely low initial target concentration ratio of approximately 4.7%. It successfully maintained a high recovery rate of 92% for 15 µm particles at the first stage sorting port. This effectively enriched the target concentration by over 2 fold, demonstrating excellent rare microparticle sorting and purification capabilities. During the second stage sorting phase, the system achieved extremely high separation purities, with the 10 µm target purity reaching 98% and the 5 µm impurity removal exceeding 99%. This purely passive flow diversion architecture operates stably with high processing throughput, providing a solid fluid dynamic foundation for the precise classification of complex mixed systems.

While the proposed cascaded dual spiral chip demonstrates excellent ternary separation for the 15, 10, and 5 µm mixture, specific limitations warrant consideration. Adapting this geometry for other size combinations necessitates proportional scaling of the channel cross section to maintain the essential blockage ratio (*a*/*D*_h_ > 0.07) and dynamic force balance. Furthermore, extreme concentration ratios, such as 1:100:100, induce severe particle crowding that disrupts focusing streamlines, requiring prior sample dilution to ensure collection purity. Moreover, exceeding the optimal flow rate disrupts the force balance by causing the Dean drag to overpower the inertial lift force, thereby deteriorating separation resolution and risking PDMS deformation and fluid leakage under elevated operational pressure. Additionally, real biological samples introduce inherent complexities. For example, circulating tumor cells exhibit a broad size distribution and cellular deformability, which may cause the focusing band to broaden and deviate from the original equilibrium position, ultimately degrading the sorting performance. To address these challenges, preventing sample adhesion and channel clogging is critical during the experimental preparation stage, and recalibrating the optimal flow rate may be necessary to maximize the sorting efficiency. Future work will focus on exploring dimensionally scalable models and integrating intelligent feedback mechanisms to accommodate a broader spectrum of particle sizes and highly complex clinical samples.

This cascaded chip serves as a highly promising microfluidic preprocessing front end module. It holds extremely broad application prospects for future highly integrated microsystems. The high purity and continuously output microparticle stream can be seamlessly connected to downstream micro nano sensing platforms. This enables full chain analysis from the physical purification of complex samples to terminal detection within an extremely small physical footprint. Consequently, this technology is expected to significantly enhance the clinical and industrial translational value of microsystems and nanoengineering. It will be particularly impactful in fields such as high throughput screening of complex microparticles and biochemical point of care testing.

## Data Availability

The data presented in this study are available on request from the corresponding author.

## Supplementary Materials

The following supporting information can be downloaded at https://www.mdpi.com/article/10.3390/mi17040469/s1: Video S1: Trajectory of 15 µm microparticles at the first stage bifurcation; Video S2: Trajectory of 10 µm microparticles at the terminus of the second stage; Video S3: Trajectory of 5 µm microparticles at the terminus of the second stage.
